# Changes in Phospholipid/Ceramide Profiles and Eicosanoid Levels in the Plasma of Rats Irradiated with UV Rays and Treated Topically with Cannabidiol

**DOI:** 10.3390/ijms22168700

**Published:** 2021-08-13

**Authors:** Wojciech Łuczaj, Anna Jastrząb, Maria do Rosário Domingues, Pedro Domingues, Elżbieta Skrzydlewska

**Affiliations:** 1 Department of Analytical Chemistry, Medical University of Bialystok, Mickiewicza 2d, 15-222 Bialystok, Poland; anna.jastrzab@umb.edu.pl (A.J.); elzbieta.skrzydlewska@umb.edu.pl (E.S.); 2 Mass Spectrometry Center, LAQV, Department of Chemistry, Campus Universitário de Santiago, University of Aveiro, 3810-193 Aveiro, Portugal; mrd@ua.pt (M.d.R.D.); p.domingues@ua.pt (P.D.); 3 CESAM, Department of Chemistry, Campus Universitário de Santiago, University of Aveiro, 3810-193 Aveiro, Portugal

**Keywords:** lipidomics, cannabidiol, ceramides, eicosanoids, phospholipids, plasma, rats, UV irradiation

## Abstract

Chronic UV radiation causes oxidative stress and inflammation of skin and blood cells. Therefore, in this study, we assessed the effects of cannabidiol (CBD), a natural phytocannabinoid with antioxidant and anti-inflammatory properties, on the phospholipid (PL) and ceramide (CER) profiles in the plasma of nude rats irradiated with UVA/UVB and treated topically with CBD. The results obtained showed that UVA/UVB radiation increased the levels of phosphatidylcholines, lysophospholipids, and eicosanoids (PGE2, TxB2), while downregulation of sphingomyelins led to an increase in CER[NS] and CER[NDS]. Topical application of CBD to the skin of control rats significantly upregulated plasma ether-linked phosphatidylethanolamines (PEo) and ceramides. However, CBD administered to rats irradiated with UVA/UVB promoted further upregulation of CER and PEo and led to significant downregulation of lysophospholipids. This was accompanied by the anti-inflammatory effect of CBD, manifested by a reduction in the levels of proinflammatory PGE2 and TxB2 and a dramatic increase in the level of anti-inflammatory LPXA4. It can therefore be suggested that topical application of CBD to the skin of rats exposed to UVA/UVB radiation prevents changes in plasma phospholipid profile resulting in a reduction of inflammation by reducing the level of LPE and LPC species and increasing antioxidant capacity due to upregulation of PEo species.

## 1. Introduction

UV phototherapy is currently one of the most widely used treatments for skin diseases [1,2]. Although long-term skin exposure to UV radiation has a therapeutic effect, it also causes redox imbalance, leading to the induction of oxidative stress in cells [3]. In such a situation, the metabolism of the main cellular components, proteins, and phospholipids is significantly altered. Oxidative stress promotes oxidative modifications in phospholipids in ROS-dependent reactions, leading to alteration of the levels of phospholipids as well as increased activity of enzymes responsible for the oxidative metabolism of phospholipids, which induces mechanisms promoting the formation of lipid mediators, such as eicosanoids or signaling molecules [4]. In order to maintain conditions close to physiological, a common strategy is to search for compounds, in particular of natural origin, having antioxidant properties and, in this case, capable of protecting phospholipids.

In recent years, research has been conducted on the effects of cannabidiol (CBD), a phytocannabinoid found in *Cannabis sativa* L., which does not have psychoactive properties [5] but exhibits antioxidant and anti-inflammatory properties on the skin and skin cells [6,7]. In various experimental models, ranging from a typical in vitro experiment using cell cultures to ex vivo experiments using cells isolated from the skin of patients, and animal studies, the effects of cannabidiol have been extensively evaluated [8,9,10,11,12,13]. CBD has been shown to restore the redox balance in cells exposed to external physicochemical factors and in skin cells of psoriatic patients [14,15,16]. Research results so far have shown that CBD contributes to the reduction of ROS levels and the normalization of antioxidant parameters. In addition, it affects the level of endocannabinoids and supports them in the activation of G-protein-coupled receptors, including cannabinoid receptors as well as TRPVs and PPARs, which are involved in the regulation of ROS and proinflammatory cytokines levels, including TNFα [17,18,19].

The resulting mechanisms described above is the protection against the oxidation of macromolecules essential to the organism, such as DNA, proteins, and lipids [17,20]. Since CBD is a lipophilic molecule, its action is particularly observed in the lipid sphere. As such, it can be used for the protection of phospholipids against ROS-dependent peroxidation of polyunsaturated fatty acids. This process leads to the formation of reactive, low molecular weight electrophilic oxidation products that include α,β-unsaturated aldehydes, which modify the structure of peptides and proteins [9]. In addition, CBD reduces the level of ROS, normalizes the activity of enzymes involved in the enzymatic metabolism of phospholipids, and prevents changes in the level of eicosanoids, which act as both proinflammatory and anti-inflammatory mediators [15]. CBD applied to the skin has been shown to prevent changes in the protein and phospholipid profile of keratinocytes and fibroblasts of rats whose skin was exposed to UV radiation [8,11].

Although many studies have been carried out to date, the effect of cannabidiol applied topically to the skin on the systemic lipid metabolism observed in the blood has not been assessed. However, the presence of CBD in the blood plasma of rats following topical application of this phytocannabinoid on their skin confirmed in our recent study [21] prompted the evaluation of the effect of CBD on changes in the profile of phospholipids and ceramides in the blood plasma of rats exposed to UV radiation, which was the aim of this study.

## 2. Results

An important observation that provides the basis for further analysis of lipidomic changes at the systemic level was our recent study, which revealed that CBD applied chronically to the skin of nude rats enters the bloodstream [21]. The concentration of cannabidiol in the blood plasma of the animals, determined using LC–MS/MS, was 40.7 ± 7.8 pmol/mL in the group of rats to which CBD was applied (CBD group); 44.5 ± 9.3 pmol/mL in the group of rats irradiated with UVA and treated with CBD (UVA+CBD group); and 25.8 ± 6.2 pmol/mL in the group of animals irradiated with UVB and treated with CBD (UVB+CBD group), whereas a statistically significant difference, namely a decrease in the level of this fitocannabinoid, was observed only in the plasma of animals irradiated with UVB and treated with CBD. These results show that the CBD present in the blood may affect systemic lipid metabolism.

The main objective of the present study was to verify the efficacy of CBD in vivo by evaluating the changes in plasma phospholipid and ceramide profiles resulting from topical application of CBD to the skin of nude rats exposed to UV radiation. Hence, the HILIC–LC–MS/MS platform was applied to characterize changes in the plasma phospholipid profile, while the RP–HPLC–MS/MS technique was used to investigate the alterations in ceramide classes. We performed the identification and the relative quantification of PL species belonging to seven different classes: phosphatidylcholine (PC), phosphatidylethanolamine (PE), phosphatidylserine (PS), phosphatidylinositol (PI), lyso-PC (LPC), lyso-PE (LPE), and sphingomyelin (SM). The complete list of the 99 PL species (corresponding to the most abundant species in all identified classes) that were identified and quantified after MS and MS/MS analysis of each sample can be found in Supplementary Tables S1 and S2. As a result of ceramide profiling, we identified the 29 most abundant ceramide species containing nonhydroxy fatty acid [N], and two sphingoid bases (dihydrosphingosine [DS] and sphingosine [S]), belong to two different classes, namely CER[NS] and CER[NDS], which are listed in Supplementary Tables S3 and S4 (Supplementary Materials). The set of identified CER species includes ceramides with an odd carbon chain. These CERs contain mainly heptadecanoic acid (C17:0), nonadecanoic acid (C19:0), tricosylic acid (C23:0), and pentacosanoic acid (C25:0). MS data confirming their identification are provided in the Supplementary Materials (Figures S1–S4).

**Figure 1:**
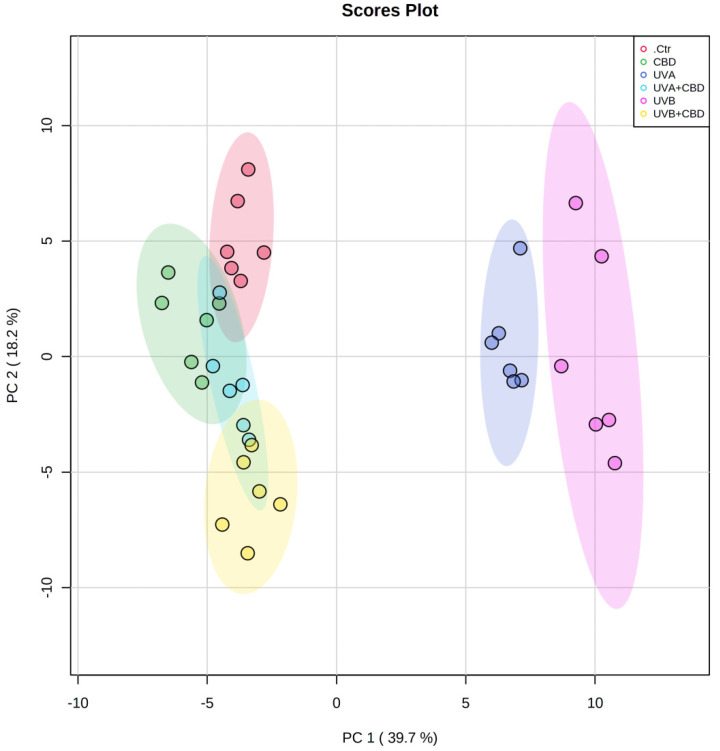
Two-dimensional principal component analysis (2D PCA) scores plot of the relative phospholipid content of control (Ctr) and UVA-irradiated rats (increasing doses from 0.5 to 5 J/cm^2^ for 4 weeks) or UVB-irradiated rats (increasing doses from 0.02 to 2 J/cm^2^ for 4 weeks). These animals have not been treated (Ctr, UVA, and UVB) or have been treated topically with CBD (CBD, UVA+CBD, and UVB+CBD) (2.5 g CBD in 100 g petrolatum).

We used multivariate and univariate statistical analyses to identify significant changes in the profiles of phospholipids and ceramides between the groups analyzed. Autoscaled data were subjected to principal component analysis (PCA) to show trends in the clustering of experimental groups (Figure 1). The two-dimensional principal component analysis (2D PCA) scores plot obtained for the phospholipid data set demonstrated that the samples were clustered into five distinct groups: control, CBD, UVA, UVB, and UVB+CBD, while the UVA+CBD group was not discriminated from control (Ctr) and CBD groups (Figure 1). Some overlapping of the confidence curves was observed between the CTR and the CBD groups. The PCA model captured 57.9% of the total variance. The variation between the groups is more pronounced on the PC1 component (39.7%), which accounts for the greatest variation that allows the discrimination of plasma samples irradiated with UVA or UVB from the other groups. The PC2 component (18.2%) mainly describes the variation between the groups of rats irradiated with UVA or UVB and treated with CBD and groups of animals treated with CBD or Ctr. The PCA plot shows that the groups of rats irradiated with UVA and UVB are scattered in the right region of the plot and separated from the other experimental groups, which were scattered in the left region of the plot (Figure 1).

Considering good discrimination of UVA+CBD and UVB+CBD groups from UVA and UVB groups, which were scattered in the opposite region of the plot and close to the Ctr and CBD groups, this shows that the changes in plasma phospholipid profiles induced by the treatment with CBD of the rats irradiated with UVA/UVB made them more similar to the phospholipid profiles of the Ctr and CBD groups.

**Figure 2:**
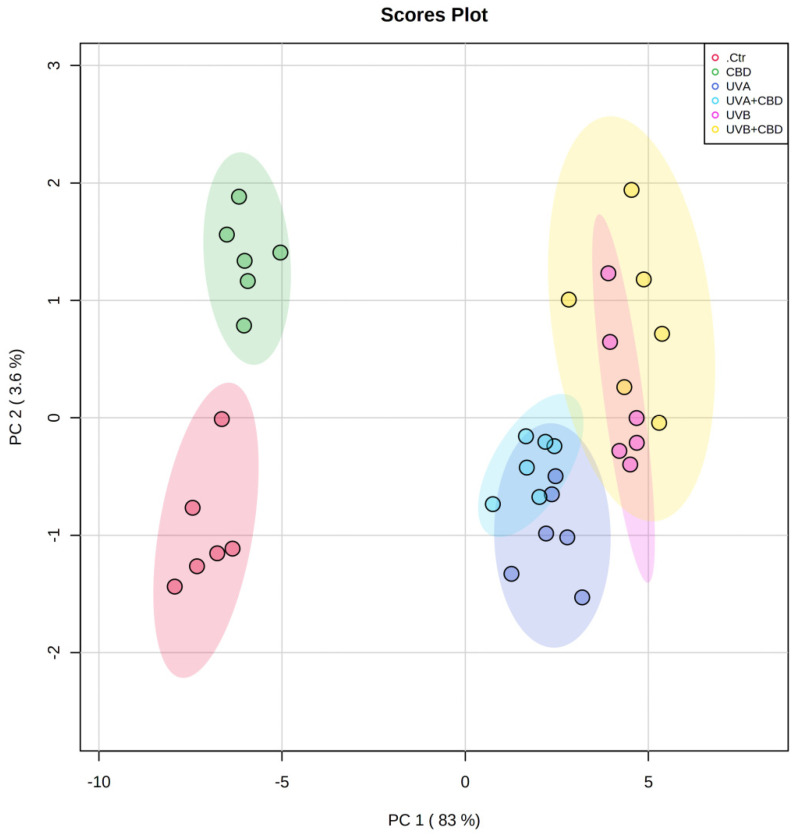
Two-dimensional principal component analysis (2D PCA) scores plot of the relative ceramide content of control (Ctr) and UVA-irradiated rats (increasing doses from 0.5 to 5 J/cm^2^ for 4 weeks) or UVB-irradiated rats (increasing doses from 0.02 to 2 J/cm^2^ for 4 weeks). These animals have not been treated (Ctr, UVA, and UVB) or have been treated topically (CBD, UVA+CBD, and UVB+CBD) (2.5 g CBD in 100 g petrolatum).

The 2D PCA plot constructed for data obtained from ceramide profiling presents the analyses describing 86.6% of the total variance, including PC1 (83%), as a major discriminating component, and PC2, which accounts for 3.6% (Figure 2).

In the PCA, PC1 mainly explains the variation between the cluster of plasma samples from control rats (Ctr) and rats that were topically treated with CBD, which were separated from each other and scattered in the left region of the plot. In the right region of the plot, we can observe four poorly separated groups, namely UVA, UVB, UVA+CBD, and UVB+CBD, although clustering can be observed depending on the treatment (UVA or UVB).

**Figure 3:**
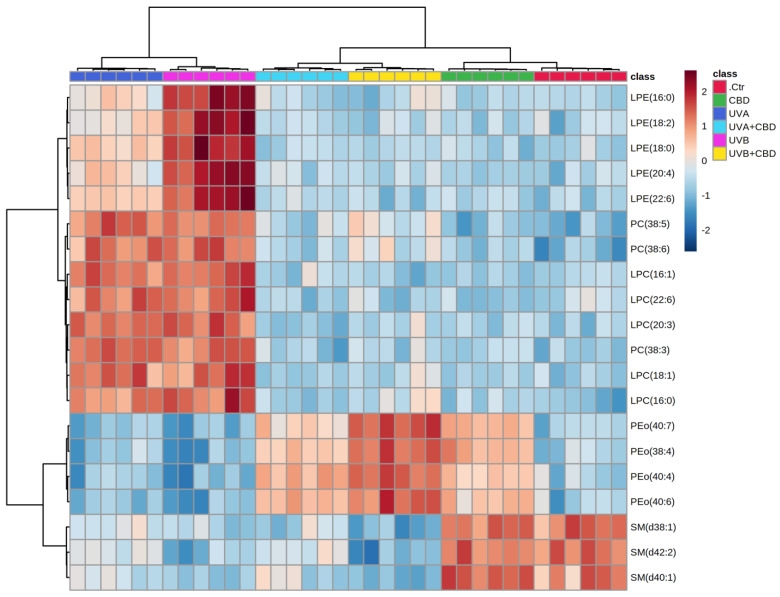
Two-dimensional hierarchical clustering heat map of the 20 most discriminating phospholipid species (according to one-way ANOVA) identified in the plasma of control (Ctr) and rats irradiated with UVA (increasing doses from 0.5 to 5 J/cm^2^ for 4 weeks) or rats irradiated with UVB (increasing doses from 0.02 to 2 J/cm^2^ for 4 weeks). These animals have not been treated (Ctr, UVA, and UVB) or have been topically treated with CBD (CBD, UVA+CBD, and UVB+CBD) (2.5 g CBD in 100 g petrolatum). Levels of relative abundance are indicated on the color scale, with numbers indicating the fold difference from the grand mean. The clustering of the sample groups is represented by the dendrogram on the top. The clustering of individual phospholipid species with respect to their similarity in the change in relative abundance is shown by the dendrogram to the left. Abbreviations: CBD, cannabidiol.

**Figure 4:**
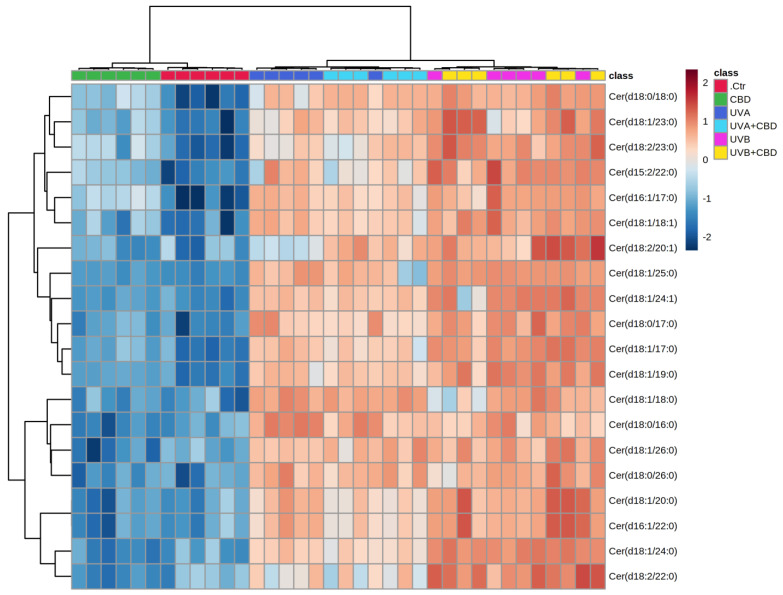
Two-dimensional hierarchical clustering heat map of the 20 most discriminating ceramide species (according to one-way ANOVA) identified in the plasma of control (Ctr) and UVA-irradiated rats (increasing doses from 0.5 to 5 J/cm^2^ for 4 weeks) or UVB-irradiated rats (increasing doses from 0.02 to 2 J/cm^2^ for 4 weeks). These animals have not been treated (Ctr, UVA, and UVB) or have been topically treated with CBD (CBD, UVA+CBD, and UVB+CBD) (2.5 g CBD in 100 g petrolatum). Levels of relative abundance are indicated on the color scale, with numbers indicating the fold difference from the grand mean. The clustering of the sample groups is represented by the dendrogram on the top. The clustering of individual ceramide species with respect to their similarity in the change of relative abundance is represented by the dendrogram to the left. Abbreviations: CBD, cannabidiol.

Univariate analysis was used to create a two-dimensional hierarchical clustering heat map using the 20 major species of phospholipids (Figure 3) and ceramide (Figure 4), according to one-way ANOVA *p* values.

The primary split in the upper hierarchical dendrogram shows that the samples were clustered independently in three main groups, in the phospholipids and ceramides analyses (Figure 3 and Figure 4). Clustering of individual lipid species (with respect to their similar expression changes) shows that they cluster into four main groups of phospholipids as well as ceramide species. In the case of phospholipids, the first group consisted of LPE species, while the second group consisted of PC and LPC. However, the third group consisted of four PEo species and the fourth group included three species of SM, namely SM(d38:1), SM(d40:1), and SM(d42:2). In the case of ceramides, three clustered groups of ceramides were composed mainly of CER[NS] and a few CER[NDS] species, while the fourth cluster was composed only of CER[NS] (Figure 4).

In the next step, we performed univariate analysis (one-way ANOVA and Tukey’s post hoc tests) to evaluate the variation in the relative abundance of phospholipid and ceramide species under the experimental conditions.

### 2.1. UVA and UVB Effects on Plasma Phospholipid and Ceramide Profiles

Comparison of the plasma phospholipid profiles of rats exposed to UVA or UVB with those of control rats showed significant upregulation of lysophospholipids, including LPE and LPC species, which was most pronounced in the UVB group (Table 1 and Figure 3).

**Figure 5:**
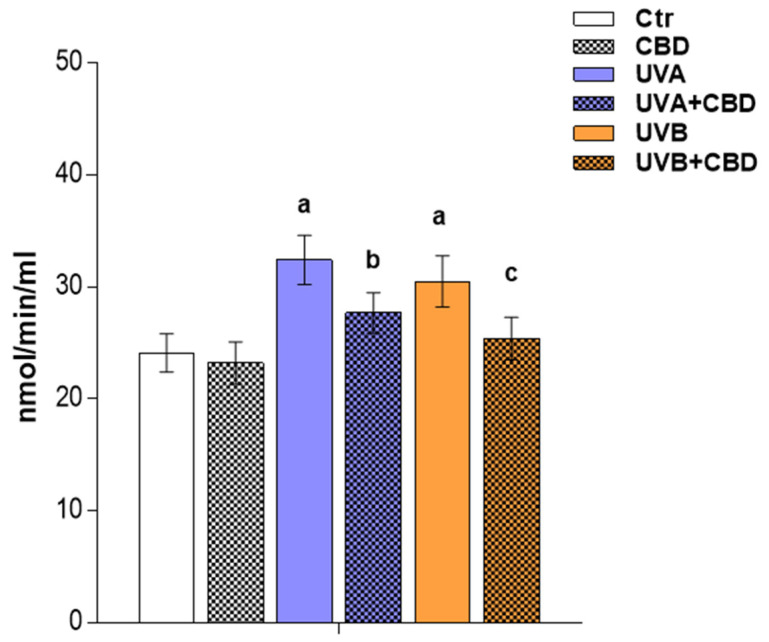
Activity of phospholipase A_2_ (PLA2) in the plasma of control rats (Ctr) and rats irradiated with UVA/UVA (UVA in increasing doses from 0.5 to 5 J/cm^2^ for 4 weeks; UVB in increasing doses from 0.02 to 2 J/cm^2^ for 4 weeks) treated and not treated with CBD (2.5 g CBD in 100 g petrolatum). Values are mean ± SD, *p* < 0.05; a—significantly different from control; b—significantly different from the rats irradiated with UVA; c—significantly different from the rats irradiated with UVB.

The observed upregulation of lyso-PC was accompanied by an increase in the relative content of the phosphatidylcholine species, namely PC(38:5), PC(38:6), and PC(38:3) in the two groups of irradiated rats. The upregulation observed in lysophospholipids in the plasma of rats exposed to UVA as well as UVB radiation was compatible with an increase in PLA_2_ activity in the plasma of these animals (Figure 5).

In contrast, the sphingomyelin species SM(d42:2), SM(d38:1), and SM(d40:1) tended to be downregulated in the plasma of the two groups of rats exposed to UVA and UVB. However, no significant changes were observed in PEo species, except for PEo(40:4), the relative abundance of which was lower in the plasma of UVB-irradiated rats compared to control animals. In addition to the indicating changes in phospholipids, a general trend toward an increase in CER species was observed in the plasma of rats irradiated with both types of UV light. A more pronounced upregulation was noted for the animals exposed to UVB, in which all relevant CER species were more upregulated compared to those irradiated with UVA (Table 2 and Figure 4).

### 2.2. Effects of Topical Application of CBD on the Plasma Phospholipid and Ceramide Profiles

Based on the results obtained for the group of rats treated topically with CBD, we found that the upregulation of several species of ether-linked phosphatidylethanolamines (PEo) and ceramides (Table 1 and Table 2, Figure 3 and Figure 4) was the main effect of topical application of this phytocannabinoid in the plasma of nonirradiated rats.

However, by evaluating the data (UVA, UVB, UVA+CBD, UVB+CBD), we found that CBD was responsible for a general trend to reverse the changes induced by the two types of UV light in the plasma phospholipid profile of rats exposed to UVA and UVB radiation. This was observed for all relevant lysophospholipids, LPE, and LPC. The relative content of these species was significantly downregulated in the plasma of rats irradiated with UV and treated with CBD compared to animals exposed to UV light (Table 1 and Figure 3). Moreover, the relative abundances of LPE and LPC in these groups did not differ significantly from those in control rats. The changes observed in downreglation of lyso-PL can be correlated with phospholipase A2 (PLA_2_) activity, which was significantly decreased in the plasma of rats irradiated with UV after topical application of CBD (Figure 5).

In contrast to the reported tendency to lower the relative content of lysophospholipids, a significant upregulation of all relevant ether-linked phosphatidylethanolamine species PEo was observed in rats irradiated with UVA and UVB after CBD application. This effect of the CBD treatment was most pronounced in the plasma of animals exposed to UVB, where the upregulation of PEo was more significant compared to the UVA group (Table 1 and Figure 3). Moreover, no significant differences were found in the relative content of sphingomyelins in the groups of rats treated with UVA and UVB after CBD treatment compared to animals exposed to UVA or UVB radiation. Likewise, no significant difference was observed after treatment with CBD in the plasma ceramide profiles of rats treated with UVA and UVB compared to the UVA and UVB groups. All ceramide species were more abundant in UVA+CBD and UVB+CBD groups compared to the Ctr group, and their relative levels were similar to those observed in UVA and UVB groups (Table 2 and Figure 3).

### 2.3. Effects of UVA or UVB and CBD Topical Application on the Level of Eicosanoids

**Figure 6:**
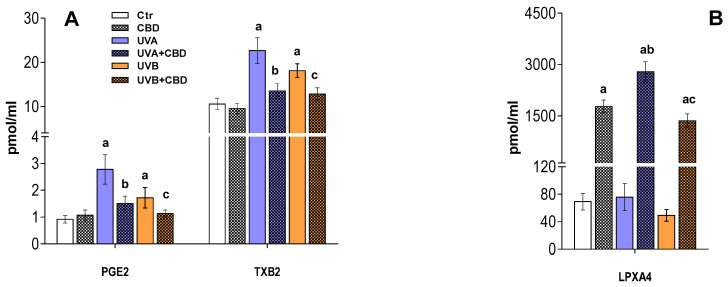
Changes in the level of inflammatory eicosanoids (**A**) and anti-inflammatory eicosanoids (**B**) in the plasma of control rats (Ctr) and rats irradiated with UVA/UVB (UVA in increasing doses from 0.5 to 5 J/cm^2^ for 4 weeks; UVB in increasing doses from 0.02 to 2 J/cm^2^ for 4 weeks) treated and untreated with CBD (2.5 g CBD in 100 g petrolatum). Values are mean ± SD, *p* < 0.05; a—significantly different from the control; b—significantly different from the rats irradiated with UVA; c—significantly different from the rats irradiated with UVB.

Our results also confirmed that topical UV radiation and topical application of CBD significantly affect plasma levels of eicosanoids (Figure 6). We found increased levels of proinflammatory eicosanoids, prostaglandin PGE_2_, and thromboxane TXB_2_ in the plasma of rats exposed to UVA and UVB, while the content of the anti-inflammatory lipoxin LPXA_4_ was similar to that of plasma from the control rats (Figure 6).

Our results also confirmed that topical application of CBD significantly affects plasma levels of eicosanoids in rats irradiated with UV. However, the most significant effects of CBD were in the level LPXA_4_, which was significantly increased in the plasma of rats treated with CBD, as well as those treated with CBD and exposed to UVA/UVB light. In contrast, the levels of proinflammatory eicosanoids (PGE_2_, TXB_2_) were elevated in the plasma of rats exposed to UVA/UVB light and significantly reduced in the plasma of rats treated with CBD and irradiated with UVA/UVB (Figure 6).

## 3. Discussion

UV radiation is one of the main environmental factors that cause damage to skin cells [22,23]. Epidermal cells, including the most abundant keratinocytes, are particularly exposed to UVB radiation, while the most abundant cells in the dermis, the fibroblasts, are mainly exposed to UVA radiation. To date, our studies investigating the effects of UV radiation and cannabidiol on keratinocytes isolated from the rat skin exposed to the above factors in vivo have shown significant changes in the phospholipids’ profiles. These changes included an upregulation of phosphatidylcholines, lysophosphatidylcholines, and phosphatidylethanolamines resulting from UV exposure and a decrease in sphingomyelin content with an increase in phosphatidylethanolamines and phosphatidylserines in animals treated with CBD [24]. However, this study did not answer the question of whether topical application of cannabidiol affects only skin cells or causes metabolic changes throughout the body. To date, the effects of CBD on various tissues have been assessed after application of this phytocannabinoid via injection, steam inhalation, or oral administration [25,26,27,28,29]. We showed that CBD applied topically to the skin is absorbed into the bloodstream [21], suggesting that this route might have an effect on the plasma phospholipid and eicosanoids (their metabolites) content. As such, we decided to investigate the changes in plasma lipid profile resulting from exposure to UV radiation and topical treatment with CBD.

Although acute and chronic exposure to UV radiation has been shown to impair antioxidant defense and increase levels of oxidative stress biomarkers not only in the skin but also in the liver and blood plasma of hairless mice and nude rats [8,24,30,31], so far, no studies have assessed the changes in plasma profiles of phospholipids or ceramides after chronic exposure of animals to UV radiation. The results of this study show that exposure of nude rats to UVA or UVB radiation induced changes in their plasma phospholipid profile, namely upregulation of phosphatidylcholine (PC) species as well as lysophospholipid species such as LPC and LPE. A similar direction of changes was observed in keratinocytes from nude rats irradiated with UVA or UVB, in which we found significantly elevated PCs and LPCs, but no changes in the levels of LPE species [24]. Lysophospholipids are formed as a result of the hydrolysis of phospholipids by phospholipase A2 (PLA2) [32], the activity of which, as shown in this study, is significantly increased in the blood plasma of rats exposed to UV radiation. This may, to some extent, explain the observed increase in the relative content of lysophospholipids, including LPC species. It has been shown that LPC, by inducing the activity of NADPH oxidase, increases the generation of superoxides in neutrophils, leading to an intensification of the inflammatory processes [33]. In addition, by increasing the generation of ROS, LPC activates caspase-1 [34,35,36], which plays an important role in inflammation through the activation of biologically inactive procytokines, such as IL-1β and IL-18 [37]. Furthermore, LPC can increase the generation of chemokines and increase the release of inflammatory factors, such as IL-1β, IL-8, IFN-γ, IL-6, and IL-5 [38]. Therefore, a significant increase in the relative content of LPC observed in this study may indicate a possible intensification of inflammatory processes in the blood of UV-irradiated rats, which has been also confirmed by the results of recent studies [21] and corroborated by the increase of proinflammatory eicoasanoides observed. However, in the case of LPC, the exact interpretation of the observed effects is difficult due to the rapid metabolism of these lysophospholipids into lysophosphatidic acid, which is also proinflammatory [39]. It should also be noted that although LPCs are widely regarded as proinflammatory mediators playing a key role in the pathogenesis of inflammatory diseases, a growing body of research points to the beneficial effects of LPC species in pathological conditions [40]. Most of the recent studies, unlike previous studies, have reported a decrease in plasma LPC levels in different disease states [41,42,43]. In addition, the upregulation of other lysophopspholipids observed in this study, namely LPE, may also suggest a possible induction of anti-inflammatory processes. Lysophosphatidylethanolamines containing polyunsaturated fatty acids have been shown to have anti-inflammatory properties attenuating the inflammatory response by increasing the level of LPXA_4_, which is an important anti-inflammatory lipid mediator [44]. In fact, among the relevant LPE species identified in our study, there are also LPEs containing polyunsaturated fatty acids (PUFAs), including arachidonic, linoleic, and docosahexaenoic acid. Therefore, our results may suggest the existence of systemic eicosanoid-dependent mechanisms involved in the inflammatory response resulting from exposure to UV radiation [45,46].

Despite the changes revealed in lysophospholipid levels, we found that sphingomyelin species tended to decrease in the plasma of rats irradiated with UVA and UVB. The indicated downregulation of sphingomyelins leads to an increase in ceramides (CER) level since the degradation of sphingomyelins by sphingomyelinase is one of the main mechanisms leading to the formation of ceramides [47]. In consequence, the results of this study showed a trend leading to an increase of CER species, belonging to both the CER [NS] and CER [NDS] classes. The upregulation of CERs was more pronounced in the plasma of animals exposed to UVB radiation, which is consistent with the greater reduction in the relative levels of sphingomyelins observed in the plasma of these rats. These results are similar to changes in CERs and SM previously observed in keratinocytes from the same rat species [24], which is consistent with the results of other authors who have found high levels of ceramides in keratinocytes irradiated with UVB and UVA [48,49]. Ceramides are the main lipid class responsible for improving the epidermal barrier, but they are also important lipid mediators involved in different metabolic pathways acting as signaling molecules [49]. Increased plasma levels of ceramides may be associated with inflammatory cytokines, e.g., interferon-γ, tumour necrosis factor-α (TNF-α), and interleukin-1ß, the levels of which increased after UV radiation and induced the generation of ceramides by activation of sphingomyelinase [50,51,52]. Elevated levels of ceramides have also been reported in the blood plasma of patients with hypertension, insulin resistance, or type 2 diabetes mellitus [50,51,52]. Therefore, it may be suggested that monitoring the blood plasma ceramide levels may help to predict pathological events or disease development [53,54,55]. It should be noted that the set of significant CER species identified in our study includes ceramides with odd carbon chain. Considering available data in the literature and the results of our research, it appears that the most possible origin of odd chain fatty acid was the rat’s diet. Dietary odd-chain saturated fatty acids are present in trace levels in dairy fat and some fish and plants. However, rodent studies suggest that odd-chain fatty acids are synthesized endogenously from gut-derived propionate [56]. It has been also revealed that propionate increases with dietary fiber consumption [57]. Therefore, we may speculate that the feed pellet that rats ate for four weeks, as their reach source of fiber, resulted in enhanced odd-chain fatty acids synthesis. This may explain to some extent the presence of decent number of CER species containing odd-chain fatty acids in the plasma of rats used in our study.

Since we confirmed the presence of CBD in the plasma of rats treated topically with this phytocannabinoid, the results presented in this study provide a first overview of the changes in the profile of phospholipids and ceramides in the plasma of UV-irradiated rats after topical application of this phytocannabinoid. Comparison of changes in plasma phospholipid profiles of rats irradiated with UVA or UVB and rats irradiated with UVA/UVB but treated topically with CBD showed a general tendency to prevent metabolic disturbances caused by both types of UV radiation. This tendency was observed in all relevant lysophospholipids (LPE and LPC), the relative content of which was significantly reduced in the plasma of UV-irradiated and CBD-treated rats compared to animals exposed only to UV. Moreover, the relative abundance of LPE and LPC in these groups did not differ significantly from control rats. The changes shown may be a significant new argument confirming the anti-inflammatory effect of CBD, especially considering the significant increase in the level of ceramide species. On the other hand, the data in the literature show that ceramide, by the formation of channels in the mitochondrial outer membrane, leads to the release of cytochrome c in the cytoplasm, leading to the intensification of cell apoptosis [58].

In contrast to the reported tendency to lower lysophospholipid levels, our study showed a significant upregulation of all relevant ether-linked phosphatidylethanolamine species (PEo) after topical application of CBD to the skin of nude rats irradiated with UVA or UVB. This effect of the CBD treatment was most pronounced in the plasma of animals exposed to UVB, where the upregulation of PEo was more significant compared to the UVA group. Interestingly, the upregulation of several species of ether-linked phosphatidylethanolamines (PEo) and CER was also observed in the blood plasma after topical application of CBD to the skin of control rats. The upregulation of CER species in plasma may be associated with a CBD-mediated induction of mechanisms leading to the generation of CER in keratinocytes or other skin cells, as has been suggested [24]. However, an increase in PEo species may be associated with different mechanisms, as these lipid species are essential in inflammation as precursors of arachidonate which is metabolized to inflammatory lipid mediators such as thromboxanes, leukotrienes, or prostaglandins [59,60]. Our results show a decrease in the levels of prostaglandin E_2_ (PGE_2_) and thromboxane B_2_ (TxB_2_) in the plasma UV-irradiated rats after treatment with CBD, compared to the untreated animals. On the other hand, CBD caused a significant increase in the level of lipoxin A_4_ (LPXA_4_), an anti-inflammatory lipid mediator strongly associated with the resolution of inflammation [61]. The changes shown in eicosanoid levels suggest a multidirectional action of CBD in response to inflammation. Therefore, the observed significant upregulation of PEo in the plasma of rats irradiated with UV after topical application of CBD can be explained to some extent when considered as a response to inflammatory processes induced by the exposure of animals to UV radiation. The results obtained are consistent with the results of other studies showing that CBD has a clear anti-inflammatory effect, especially in the case of counteracting the effects of UV radiation [8,14]. It also seems extremely important to confirm the antioxidant properties of this phytocannabinoid, associated with an increase in the content of ether phospholipids, which are characterized by the ability to eliminate oxygen radicals, most often generated under pathological conditions [62,63,64]. It is believed that since ether-bound phospholipids are more susceptible to oxidation than ester-bound phospholipids [65], they may protect other blood components from oxidation. Thus, the elevation of PEo species in the plasma of rats exposed to UV may be associated with the antioxidant action of CBD in response to oxidative stress induced by UV radiation. CBD applied topically to the skin of nude rats irradiated with UVA/UVB induces protective mechanisms in responses to oxidative stress, such as reducing the activity of PLA2, an enzyme releasing PUFAs, which undergo oxidative fragmentation with the generation of reactive aldehydes (such as MDA, 4-HNE) and oxidative cyclization to form isoprostanes subsequently released from the phospholipid backbone. Consequently, the levels of both types of compounds were significantly reduced in the plasma of rats exposed to both UV and CBD [21]. In addition to what is mentioned above, more significant upregulation of PEo in UVB irradiated rats than in animals exposed to UVA after CBD application may be associated with the lower plasma level of CBD, which resulted from its tendency to accumulate in UVB-irradiated keratinocytes, as has been recently discussed [21].

In summary, the topical application of CBD prevented the changes in plasma phospholipid profile induced by UVA/UVB irradiation of the skin of rats. This protective effect of CBD was manifested by a significant downregulation of LPE and LPC species to a content similar to that observed in the plasma of control animals, which may indicate an anti-inflammatory effect of CBD. This observation was confirmed by changes in the level of eicosanoids, namely upregulation of the anti-inflammatory LPXA_4_ and downregulation of the proinflammatory PGE_2_ and TxB_2_, which further suggest a multidirectional anti-inflammatory effect of CBD. At the same time, a significant increase in PEo, a phospholipid species capable of eliminating ROS, may suggest an improvement in the plasma redox balance. On the other hand, the significant upregulation of several CER species in the plasma of rats treated topically with CBD suggests an involvement of this phytocannabinoid in the regulation of metabolic processes including differentiation, autophagy, apoptosis, and cellular senescence. Thus, the results of this study confirm that CBD applied topically to the skin modulates lipid metabolism, restoring the redox balance and reducing inflammation in the plasma of rats irradiated with UV. It is therefore hoped that further research will reveal the exact molecular mechanisms responsible for the observed effects of cannabidiol.

## 4. Materials and Methods

### 4.1. Reagents/Chemicals

Internal standards for phospholipids and ceramides were purchased from Avanti Polar Lipids, Inc. (Alabaster, AL, USA). All chemicals were purchased from Sigma-Aldrich Chemical Co. (St. Louis, MO, USA); all solvents were of LC–MS grade. Milli-Q water was used for all experiments, filtered through a 0.22 μm filter, and obtained using a Milli-Q Millipore system (Advantage A10, Millipore Corporation, Billerica, MA, USA).

### 4.2. Study Animal Model

Male nude rats (RH-FOXN1RNU) 8–9 weeks old with bodyweight between 260 and 302 g were used in the experiments. Rats were kept under standardized conditions (12 h light/12 h dark cycles) and fed pellets [66]. The experimental procedure was approved by the Local Ethics Committee for Animal Experiments in Olsztyn (Resolution No.37/2019 of 26 April 2019). The rats, whose skin was exposed to UVA/UVB radiation and treated with CBD applied topically to the back for four weeks, were divided into the following experimental groups:

- control (Ctr): treatment with nontoxic hydrophilic petrolatum applied topically to the back for 20 min every 12 h for four weeks;
- CBD: treatment with CBD (2.5%; *w*/*w* in petrolatum) applied topically to the back for 20 min every 12 h for four weeks;
- UVA: irradiation with UVA (increasing doses 0.5–5 J/cm^2^) every 48 h for four weeks;
- UVA+CBD: irradiation with UVA (increasing doses 0.5–5 J/cm^2^) every 48 h and treated with CBD every 12 h, as in the CBD group;
- UVB: irradiation with UVB (increasing doses 0.02–2 J/cm^2^) every 48 h for four weeks;
- UVB+CBD: irradiation with UVB (increasing doses 0.02–2 J/cm^2^) every 48 h and treated with CBD every 12 h, as in the CBD group.

At the end of the experiment, the animals were anaesthetized by inhalation of isoflurane and sacrificed by cardiac excision. Blood was collected in EDTA tubes coated with butylhydroxytoluene (BHT) as an antioxidant. In order to obtain plasma, the blood samples were centrifuged at 3000× *g* at 4 °C for 20 min.

#### Determination of the Level of CBD in the Plasma

The level of CBD was determined using LC–MS/MS (LC-MS 8060, Shimadzu, Kyoto, Japan) as described previously [67]. The solid-phase extraction (SPE) technique was applied to sample preparation. From each sample, 5 µL of the sample was loaded onto an Agilent Poroshell 120 EC-C18 analytical column (3.0 × 150 mm; 2.7 µm particle size) maintained at 18 °C. The mobile phase consisted of 0.1% formic acid in water (solvent A) and 0.1% formic acid in acetonitrile (solvent B) was delivered at a flow rate maintained at 0.8 mL × min^−1^. Gradient elution was applied in the method as follows: 70% B to 80% B in 5 min; 80% B to 88% B in 10 min; 88% B to 100% B over 6 min. and hold for 4 min; 100% B to 70% B within 1 min. and hold at 70% B for 4 min to re-equilibrate the column before the next sample analysis. All analyses were performed using electrospray ionization (ESI) positive mode and quantification was performed using multiple reactions monitoring (MRM) mode. CBD-d_9_ was used as an internal standard. The transitions from the precursor to the product ion were as follows: *m/z* 315.1→193.0 for CBD; and *m*/*z* 324.1→202.2 for CBD-d_9_. CBD levels were expressed in nmol/mL.

### 4.3. Lipidomic Analysis

#### 4.3.1. Extraction of Lipids and Quantification of Phospholipid Content

Lipid extracts from plasma samples were obtained using the modified Folch method [68]. The phospholipid content of each lipid extract was calculated by the colorimetric phosphorus assay [69]. All experimental procedures concerning lipid extraction and phospholipid quantification described in detail in previously published studies [11,70].

#### 4.3.2. Phospholipid Profiling by Hydrophilic Interaction Liquid Chromatography Coupled with High-Resolution Tandem Mass Spectrometry (HILIC–MS/MS) 

Phospholipids were separated by hydrophilic interaction liquid chromatography using a UPLC system (Agilent 1290; Agilent Technologies, Santa Clara, CA, USA) coupled with a QTOF mass spectrometer (Agilent 6540; Agilent Technologies, Santa Clara, CA, USA). Internal standards of PC(14:0/14:0), LPC(19:0), PE(14:0/14:0), PI(16:0/16:0), and PS(14:0/14:0) were used for the quantification and assessment of the ions variations. The mixture composed of solvent A [ACN/MeOH/water 50:25:25 (*v*/*v*/*v*) with 1 mM ammonium acetate] and solvent B [ACN/MeOH 60:40 (*v*/*v*) with 1 mM ammonium acetate] was used as mobile phase. The gradient elution was applied started with 0% of A, increased linearly to 100% of A within 20 min and held for 15 min, then returned to 0% of A in 10 min. Twenty-five μg of each phospholipid extract corresponding to a volume of 10 μL was mixed with 90 μL of mobile phase (60% of A and 40% of B). A volume of 10 μL of diluted sample was loaded into the Ascentis^®^ Si column (15 cm × 1 mm, 3 μm, Sigma-Aldrich) with the mobile phase flow rate of 40 μL per min. The QTOF mass spectrometer operated using a negative-ion mode (electrospray voltage, −3000 V) with a capillary temperature of 250 °C and sheath gas flow of 13 L/min. The data-dependent acquisition mode (DDA) was used for data collecting in the range of *m*/*z* 100–1500 with a fixed collision energy of 35 eV. The LPE, PE, PI, and PS species were analyzed as [M − H]^−^ ions, while LPC, PC, and SM species were analyzed as [M + CH_3_COO]^−^ adducts. Data acquisition was carried out with the use of Mass Hunter data software (version B0.8.0, Agilent Technologies, Santa Clara, CA, USA). The relative content of each phospholipid ion species was achieved by normalization the area of each peak to the peak area of the corresponding internal standard. The retention times and obtained MS/MS spectra were the basis for phospholipid identification.

#### 4.3.3. Ceramide Profiling by Reversed-Phase Chromatography Coupled with High-Resolution Tandem Mass Spectrometry RPLC-MS/MS Analysis of Ceramides

An Agilent UPLC–ESI–QTOF–MS system (Agilent 1290; Agilent 6540; Agilent Technologies, Santa Clara, CA, USA) was used to characterize the CER profiles. The mobile phase was composed of solvent A (water with 20 mM ammonium formate pH 5) and solvent B (methanol). Initially, 70% of B was held isocratically for 1 min, followed by a linear increase to 100% of B within 75 min, and return to initial conditions in 5 min. The ceramides were separated on an RP C18 column (Acquity BEH Shield 2.1 × 100 mm; 1.7 μm; Waters, Milford, MA, USA) with a flow rate of 0.5 mL/min. The QTOF mass spectrometer was operated in positive ion mode (electrospray voltage 3.5 kV) with a capillary temperature of 300 °C and a sheath gas flow rate of 8 L/min. The data was collected in DDA mode. Identification of ceramide species was based on the presence of the [M+H]^+^ molecular ion, retention time, and characteristic fragmentation patterns observed in MS/MS spectra, which was previously described in detail [71].

#### 4.3.4. Data Processing

The filtering, peak detection, alignment, and integration as well as the assignment of each phospholipid species was carried out by the MZmine 2.30 software for the data obtained [72]. 

#### 4.3.5. Statistical Analysis

Univariate and multivariate statistical analyses were performed using Metaboanalyst version 4.0 [73]. The data obtained by MS/MS analysis were autoscaled before principal component analysis (PCA). Univariate statistical analysis was carried out using the ANOVA test with Tukey’s post hoc test with *p* < 0.05 considered statistically significant. The heatmaps were created using “Euclidean” as the clustering distance and “Ward” as the clustering algorithm. 

### 4.4. Enzymatic Phospholipid Metabolism

#### 4.4.1. Measurement of PLA_2_ Activity

The spectrophotometric method [74] was used to determine phospholipase A_2_ (PLA2-EC.3.1.1.4) activity (PLA2 Assay Kit no. 765021, Cayman Chemical Company, Ann Arbor, MI, USA). The activity was calculated by measuring the absorbance at 414 nm, using the DTNB [5,5′-dithio-bis-(2-nitrobenzoic acid)] extinction coefficient of 10.66 per mM per cm, and reported as nmol/min/mL.

#### 4.4.2. Eicosanoids Profiling

Quantification of the eicosanoids TXB_2_, PGE_2_, and LPXA_4_ was performed by LC-MS/MS in negative ESI mode (LCMS 8060, Shimadzu, Kyoto, Japan) as previously described [75]. LTB_4_-d_4_, TXB_2_-d_4_, and PGD_2_-d_4_ were used as internal standards for quantification. The eicosanoid fraction was extracted from plasma using SPE. The quantitative analysis was performed using MRM mode by monitoring the precursor to the product ion transition *m*/*z* 369.3→169.1 for TXB_2_, *m*/*z* 351.3→271.2 for PGE_2_, *m*/*z* 351.3→217.2 for LPXA_4_; 339.1→197.1 for LTB_4_-d_4_, 373.0→173.1 for TXB_2_-d_4_ and 355.0→275.3 for PGD_2_-d_4_.

#### 4.4.3. Statistical Analysis

The data are expressed as average ± SD (*n* = 6). The data were analyzed using one-way ANOVA combined with Tukey’s post hoc tests. A *p* value < 0.05 was considered significant. Statistical analyses were performed using GraphPad Prism 7 for Windows version 7.0.0 (GraphPad Software, San Diego, CA, USA).

## Figures and Tables

**Table 1:** Alteration observed in the molecular species of the 20 most discriminating phospholipid species (according to one-way ANOVA and Fisher’s LSD post hoc tests) in the plasma of control rats (Ctr) and rats irradiated with UVA (increasing doses from 0.5 to 5 J/cm^2^ for 4 weeks) or rats irradiated with UVB (increasing doses from 0.02 to 2 J/cm^2^ for 4 weeks). These animals were not treated (Ctr, UVA, and UVB) or were treated topically with CBD (CBD, UVA+CBD, and UVB+CBD) (2.5 g CBD in 100 g petrolatum);↑ upregulation; ↓ downregulation.

Phospholipid Specie	Log_2_ (Fold-Change)						
	CBD vs. Ctr	UVA vs. Ctr	UVA+CBD vs. Ctr	UVA+CBD vs. UVA	UVB vs. Ctr	UVB+CBD vs. Ctr	UVB+CBD vs. UVB
LPE(16:0)	-	1.33 ↑	-	1.15 ↓	4.47 ↑	-	4.15 ↓
LPE(18:2)	-	1.06 ↑	-	1.13 ↓	4.07 ↑	-	4.08 ↓
LPE(18:0)	-	1.69 ↑	-	1.60 ↓	4.20 ↑	-	4.12 ↓
LPE(20:4)	-	1.26 ↑	-	1.01 ↓	3.77 ↑	-	3.62 ↓
LPE(22:6)	-	1.87 ↑	-	1.59 ↓	4.58 ↑	-	4.55 ↓
PC(38:5)	-	2.11 ↑	-	1.59 ↓	2.02 ↑	-	1.11 ↓
PC(38:6)	-	1.67 ↑	-	1.29 ↓	1.77 ↑	-	1.04 ↓
PC(38:3)	-	2.09 ↑	-	1.98 ↓	2.01 ↑	-	1.67 ↓
LPC(16:1)	-	2.64 ↑	-	2.52 ↓	2.92 ↑	-	3.20 ↓
LPC(22:6)	-	1.99 ↑	-	2.29 ↓	4.58 ↑	-	2.38 ↓
LPC(20:3)	-	2.42 ↑	-	2.71 ↓	2.53 ↑	-	2.28 ↓
LPC(18:1)	-	2.47 ↑	-	2.57 ↓	2.62 ↑	-	2.22 ↓
LPC(16:0)	-	2.60 ↑	-	2.33 ↓	3.22 ↑	-	2.21 ↓
PEo(40:7)	2.11 ↑	-	1.55 ↑	1.93 ↑	-	3.50 ↑	4.12 ↑
PEo(38:4)	2.60 ↑	-	1.96 ↑	2.20 ↑	-	3.76 ↑	4.69 ↑
PEo(40:4)	1.83 ↑	-	2.40 ↑	2.83 ↑	1.47 ↓	3.55 ↑	4.59 ↑
PEo(40:6)	1.78 ↑	-	1.87 ↑	2.32 ↑	-	3.09 ↑	3.91 ↑
SM(d38:1)	-	1.63 ↓	1.89 ↓	-	2.01 ↓	2.61 ↓	-
SM(d42:2)	-	1.53 ↓	1.53 ↓	-	2.29 ↓	2.58 ↓	-
SM(d40:1)	-	1.45 ↓	1.43 ↓	-	1.99 ↓	2.26 ↓	-

**Table 2:** The alteration observed in the molecular species of the 20 most discriminating ceramide species (according to the one-way ANOVA and Tukey’s post hoc tests) in plasma of control rats (Ctr) and the rats irradiated with UVA (increasing doses from 0.5 to 5 J/cm^2^ for 4 weeks) or rats irradiated with UVB (increasing doses from 0.02 to 2 J/cm^2^ for 4 weeks). These animals were not treated (Ctr, UVA, and UVB) or were treated topically with CBD (CBD, UVA+CBD, and UVB+CBD) (2.5 g CBD in 100 g petrolatum);↑ upregulation; ↓ downregulation. (non-hydroxy fatty acid [N], dihydrosphingosine [DS], and sphingosine [S]).

CER Class	Ceramide Specie	Log_2_ (Fold-Change)						
		CBD vs. Ctr	UVA vs. Ctr	UVA+CBD vs. Ctr	UVA+CBD vs. UVA	UVB vs.Ctr	UVB+CBD vs. Ctr	UVB+CBD vs. UVB
CER[NS]	Cer(d18:1/23:0)	1.27 ↑	3.10 ↑	3.21 ↑	-	3.36 ↑	1.96 ↑	1.11 ↑
CER[NS]	Cer(d18:2/23:0)	2.32 ↑	3.96 ↑	4.05 ↑	-	5.12 ↑	5.66 ↑	-
CER[NS]	Cer(d15:2/22:0)	1.34 ↑	3.48 ↑	2.83 ↑	-	4.50 ↑	4.08 ↑	-
CER[NS]	Cer(d16:1/17:0)	2.99 ↑	5.26 ↑	4.82 ↑	-	6.08 ↑	5.48 ↑	-
CER[NS]	Cer(d18:1/18:1)	1.27 ↑	3.75 ↑	3.28 ↑	-	4.26 ↑	4.25 ↑	-
CER[NS]	Cer(d18:2/20:1)	-	1.09 ↑	2.11 ↑	1.02 ↑	2.33 ↑	2.75 ↑	-
CER[NS]	Cer(d18:1/25:0)	-	7.33 ↑	6.41 ↑	-	7.96 ↑	8.01 ↑	-
CER[NS]	Cer(d18:1/24:1)	-	2.17 ↑	2.08 ↑	-	2.77 ↑	2.52 ↑	-
CER[NS]	Cer(d18:1/17:0)	-	2.51 ↑	2.20 ↑	-	3.06 ↑	3.05 ↑	-
CER[NS]	Cer(d18:1/19:0)	-	2.26 ↑	2.22 ↑	-	2.83 ↑	2.81 ↑	-
CER[NS]	Cer(d18:1/18:0)	-	2.99 ↑	2.97 ↑	-	2.87 ↑	2.41 ↑	-
CER[NS]	Cer(d18:1/26:0)	-	2.08 ↑	2.32 ↑	-	2.38 ↑	2.66 ↑	-
CER[NS]	Cer(d18:1/20:0)	-	1.76 ↑	1.42 ↑	-	2.04 ↑	2.31 ↑	-
CER[NS]	Cer(d16:1/22:0)	-	1.77 ↑	1.41 ↑	-	2.05 ↑	2.32 ↑	-
CER[NS]	Cer(d18:1/24:0)	-	1.46 ↑	1.32 ↑	-	2.07 ↑	2.10 ↑	-
CER[NS]	Cer(d18:2/22:0)	-	0.97 ↑	-	-	1.83 ↑	1.85 ↑	-
CER[NDS]	Cer(d18:0/18:0)	1.96 ↑	3.45 ↑	4.01 ↑	-	4.11 ↑	4.46 ↑	-
CER[NDS]	Cer(d18:0/17:0)	-	2.62 ↑	2.06 ↑	-	2.92 ↑	3.05 ↑	-
CER[NDS]	Cer(d18:0/16:0)	-	2.72 ↑	2.21 ↑	-	2.38 ↑	2.01 ↑	-
CER[NDS]	Cer(d18:0/26:0)	-	2.15 ↑	2.05 ↑	-	2.07 ↑	2.24 ↑	-

## Data Availability

Data is contained within the article or supplementary material.

## Supplementary Materials

The following are available online at https://www.mdpi.com/article/10.3390/ijms22168700/s1.
