# Supplementary material for: Changes in Phospholipid/Ceramide Profiles and Eicosanoid Levels in the Plasma of Rats Irradiated with UV Rays and Treated Topically with Cannabidiol
Source: Int J Mol Sci. 2021 Aug 13;22(16):8700. doi: 10.3390/ijms22168700 (PMC8395479; doi:10.3390/ijms22168700)
Supplement: Supplementary file 1 [file ijms-22-08700-s001.zip › Supplementary materials - Tables 1-4 and Figures 1-4.pdf]

Supplementary materials

Table S1. Phospholipid molecular species identified in the plasma of nude rats used in the present study (phosphatidylcholine (PC), lyso-PC (LPC), phosphatidylethanolamine (PE), lyso-PE (LPE), phosphatidylinositols (PI), phosphatidylserine (PS), and sphingomyelin (SM)).

Phospholipid Class	m/z	Retention Time	Phospholipid Specie
**PC**	816.5778	19.08	PC(34:2)
	868.6040	19.86	PC(38:4)
	840.5737	19.26	PC(36:4)
	844.6035	19.65	PC(36:2)
	842.5903	19.53	PC(36:3)
	818.5883	19.09	PC(34:1)
	868.5568	19.81	PCp(40:10)
	864.5747	19.99	PC(38:6)
	892.6048	19.64	PC(40:6)
	866.5910	19.96	PC(38:5)
	870.6209	19.01	PC(38:3)
	844.5501	19.72	PCp(38:8)
	950.6202	19.47	PCp(46:11)
	846.6240	19.74	PC(36:1)
	792.5719	19.47	PC(32:0)
	894.6207	19.68	PC(40:5)
	888.5756	19.52	PC(40:8)
	870.5661	19.91	PCp(40:9)
	890.5891	19.64	PC(40:7)
	922.5972	19.68	PCp(44:11)
	948.6042	19.46	PCp(46:12)
	820.6091	19.15	PC(34:0)
**LPC**	554.3439	22.48	LPC(16:0)
	582.3756	21.93	LPC(18:0)
	578.3443	22.30	LPC(18:2)
	602.3441	21.68	LPC(20:4)
	580.3624	22.11	LPC(18:1)
	626.3462	21.67	LPC(22:6)
	604.3588	21.78	LPC(20:3)
	552.3307	22.79	LPC(16:1)
**PE**	766.5380	7.55	PE(38:4)
	742.5367	7.84	PE(36:2)
	738.5096	7.83	PE(36:4)
	764.5207	7.69	PE(38:5)
	762.5075	7.72	PE(38:6)
	740.5248	7.88	PE(36:3)
	716.5242	8.04	PE(34:1)
	790.5375	7.55	PE(40:6)
	794.5686	7.75	PE(40:4)
	744.5538	7.86	PE(36:1)
	768.5529	7.69	PE(38:3)
	750.5412	7.55	PEo(38:5)
	722.5106	7.60	PEo(36:5)

	748.5272	7.54	PEo(38:6)
	714.5052	7.98	PEo(34:3)
	752.5589	7.68	PEo(38:4)
	778.5768	7.51	PEo(40:5)
	746.5134	7.61	PEo(38:7)
	780.5891	7.70	PEo(40:4)
	724.5274	7.81	PEo(36:4)
	774.5438	7.45	PEo(40:7)
	776.5590	7.62	PEo(40:6)
	726.5422	7.84	PEo(36:3)
	700.5226	7.99	PEo(34:2)
**LPE**	480.3105	10.55	LPE(18:0)
	452.2770	10.80	LPE(16:0)
	476.2780	10.71	LPE(18:2)
	500.2767	10.32	LPE(20:4)
	478.2921	10.61	LPE(18:1)
	524.2756	10.38	LPE(22:6)
**PI**	915.5980	4.28	PI(40:3)
	885.5496	5.23	PI(38:4)
	917.6110	4.23	PI(40:2)
	857.5156	5.33	PI(36:4)
	953.5180	4.15	PI(44:12)
	887.5594	5.31	PI(38:3)
	955.5335	4.17	PI(44:11)
	971.6600	3.56	PI(44:3)
	973.6766	3.56	PI(44:2)
	999.6964	3.65	PI(46:3)
	943.6281	3.56	PI(42:3)
	945.6456	3.57	PI(42:2)
	969.6376	4.21	PI(44:4)
	997.6681	3.86	PI(46:4)
	779.4702	3.68	PI(30:1)
**PS**	816.5727	18.09	PS(38:1)
	868.6040	16.86	PS(42:3)
	840.5737	17.26	PS(40:3)
	844.6035	17.65	PS(40:1)
	818.5883	18.06	PS(38:0)
	864.5747	16.99	PS(42:5)
	842.5903	17.53	PS(40:2)
	866.5910	16.96	PS(42:4)
	892.6048	16.64	PS(44:5)
	870.6209	17.01	PS(42:2)
	846.6240	17.74	PS(40:0)
	894.6207	16.68	PS(44:4)
	888.5756	16.52	PS(44:7)
	916.6055	15.85	PS(46:7)
	890.5891	16.64	PS(44:6)
**SM**	845.6754	20.93	SM(d40:1)
	871.6900	19.47	SM(d42:2)
	843.6597	20.96	SM(d40:2)
	761.5823	21.13	SM(d34:1)

763.5897	21.17	SM(d34:0)
787.5974	21.06	SM(d36:2)
789.6128	21.09	SM(d36:1)
791.4909	21.04	SM(d36:0)
817.6433	20.98	SM(d38:1)

Table S2. Peak area of each phospholipid species identified in the plasma of control rats (Ctr) and rats irradiated with UVA (increasing doses from 0.5 to 5 J/cm^2 for 4 weeks) or rats irradiated with UVB (increasing doses from 0.02 to 2 J/cm^2 for 4 weeks). These animals were not treated (Ctr, UVA, and UVB) or were treated topically with CBD (CBD, UVA+CBD, and UVB+CBD) (2.5 g CBD in 100 g petrolatum). Data obtained using MZmine software (XLSX).

	A	B	C	D	E	F	G	H	I	J	K	L	M
1	Name	1o	2o	3o	11o	12o	13o	21o	22o	23o	24o	25o	26o
2	Label	.Ctr	.Ctr	.Ctr	.Ctr	.Ctr	.Ctr	CBD	CBD	CBD	CBD	CBD	CBD
3	PC(34:2)	9.837206	6.397001	4.384955	9.616255	7.997729	10.65324	8.051368	10.01775	6.545828	8.774202	11.85447	12.57312
4	PC(38:4)	9.438334	8.41969	8.922986	9.226069	7.673442	9.491436	11.3674	8.36434	9.24179	11.9491	9.075207	10.82441
5	PC(36:4)	6.254856	4.913335	5.173981	6.396456	5.085249	6.711187	6.197529	6.18651	5.038642	5.882101	8.292637	5.254707
6	PC(36:2)	6.534269	6.394469	3.873896	2.920467	5.312414	5.166606	6.182242	4.215395	5.026213	3.883274	5.057836	9.279871
7	PC(36:3)	2.367499	2.545238	1.338419	2.698337	1.924796	1.988446	2.553338	1.553325	2.075884	1.710502	2.705502	1.489102
8	PC(34:1)	3.260962	3.473866	2.897432	2.463271	2.651188	2.60712	2.027237	2.341813	1.64816	1.873046	2.54048	3.120411
9	PCp(40:10	0.70118	0.289287	0.41554	0.657675	0.570065	0.797011	0.569497	0.479621	0.463006	0.577218	0.679325	0.541243
10	PC(38:6)	1.640809	1.278705	1.580525	2.064542	1.333991	2.232492	2.077557	1.931642	1.689071	2.34824	2.303787	2.277186
11	PC(40:6)	1.929715	1.723127	1.786073	1.108969	1.568874	1.692936	1.930315	0.686886	1.569362	1.83541	1.593323	1.365996
12	PC(38:5)	1.286671	1.31918	1.144783	1.764225	1.046074	0.987262	1.487181	0.979405	1.20909	1.863058	1.39363	1.339822
13	PC(38:3)	1.412542	0.993605	1.715383	1.236806	1.148408	1.302845	1.722236	1.25325	1.400192	1.726723	1.330606	1.750429
14	PCp(38:8)	0.475191	0.162951	0.362156	0.074754	0.386334	0.656332	0.661239	0.122005	0.537593	0.099011	0.662452	0.40531
15	PCp(46:11	0.714939	0.671188	0.485489	0.589395	0.581251	0.537808	0.806606	0.683148	0.655778	0.533044	0.358363	0.532855
16	PC(36:1)	0.620611	0.865683	0.71161	0.605767	0.504562	0.51649	0.927725	0.725096	0.754248	1.132255	0.748499	1.142643
17	PC(32:0)	0.73551	0.577063	0.569003	0.714636	0.597975	0.29705	0.727767	0.559581	0.59168	0.468016	1.313261	0.538424
18	PC(40:5)	0.31673	0.693862	0.647676	0.689564	0.257504	0.567668	0.60376	0.236095	0.490862	0.857113	0.618971	0.689442
19	PC(40:8)	0.43811	0.372598	0.236535	0.515501	0.356187	0.316759	0.302342	0.366689	0.245807	0.300335	0.579092	0.260599
20	PCp(40:9)	0.157519	0.170993	0.127373	0.141065	0.128064	0.250533	0.186898	0.155357	0.15195	0.080314	0.27191	0.195425
21	PC(40:7)	0.290945	0.240959	0.19473	0.286569	0.23654	0.178171	0.276922	0.107713	0.22514	0.245202	0.362584	0.255232
22	PCp(44:11	0.221243	0.08132	0.147747	0.148346	0.179872	0.127688	0.178336	0.195577	0.144989	0.282901	0.096384	0.192736
23	PCp(46:12	0.099784	0.039105	0.08059	0.093299	0.081125	0.051096	0.046019	0.10939	0.037414	0.078915	0.094014	0.097251
24	PC(34:0)	0.365986	0.291368	0.146577	0.243643	0.29755	0.310368	0.401932	0.132234	0.326774	0.303743	0.38802	0.254428
25	LPC(16:0)	8.510884	13.86919	13.00135	11.01073	6.919418	12.04277	13.75105	19.4225	11.17972	20.10037	9.749901	15.08484
26	LPC(18:0)	8.004923	10.38019	5.752689	8.839438	6.508067	5.35755	7.905998	7.345667	6.427641	5.431131	5.988732	7.609278
27	LPC(18:2)	10.60336	4.801017	3.959165	5.491856	8.620615	5.795594	8.148117	9.441437	6.624485	5.296626	5.53466	5.191186
28	LPC(20:4)	3.81044	6.344392	2.989546	2.366538	3.097919	5.251248	2.525508	4.807424	2.053259	3.471776	3.556006	3.508554
29	LPC(18:1)	2.226535	3.179634	1.557195	2.661044	1.810191	1.877929	2.598566	2.500236	2.112655	2.084057	1.869483	2.039464
30	LPC(22:6)	0.45325	0.287871	0.302181	0.576259	0.368496	0.477151	0.310198	0.253407	0.252193	0.271459	0.468125	0.260768
31	LPC(20:3)	0.546063	0.470213	0.308665	0.275912	0.443954	0.486353	0.501696	0.519285	0.407883	0.319482	0.407158	0.551121
32	LPC(16:1)	0.495575	0.343556	0.333487	0.451843	0.402907	0.426182	0.361541	0.412354	0.293935	0.425925	0.305206	0.276085
33	LPE(18:0)	0.619557	0.60012	0.575208	1.226991	0.503705	0.63966	0.846821	0.818626	0.688472	0.510407	1.065021	0.383867
34	LPE(16:0)	0.732679	0.96495	0.86658	1.009179	0.595674	0.823919	0.798258	0.610766	0.64899	1.002178	1.301192	0.920236
35	LPE(18:2)	1.194542	1.454029	0.400302	1.17627	0.971173	0.698889	0.659256	0.999235	0.53598	1.215605	0.814928	0.870249
36	LPE(20:4)	0.642579	0.39591	0.252699	0.397629	0.522422	0.597243	0.415532	0.451467	0.337831	0.405358	0.426521	0.392899
37	LPE(18:1)	0.411221	0.429736	0.285432	0.549366	0.334326	0.543303	0.487292	0.156752	0.396172	0.577791	0.618956	0.587821
38	LPE(22:6)	0.116845	0.056319	0.123374	0.061632	0.094996	0.148128	0.133229	0.086408	0.108316	0.140766	0.151447	0.078363
39	PE(38:4)	0.632559	0.73047	0.487919	0.963499	0.514276	0.647401	1.059563	1.006374	0.861433	0.633646	1.602262	0.869447
40	PE(36:2)	0.494858	1.026253	0.322079	0.798076	0.402323	0.56168	0.652854	0.814709	0.530775	0.784824	0.608885	0.487191
41	PE(36:4)	0.781536	0.795874	0.306377	0.71858	0.635395	0.583002	0.687123	0.83796	0.558636	1.141259	1.269095	0.429287

	A	N	O	P	Q	R	S	T	U	V	W	X	Y	Z
1	Name	31o	32o	33o	34o	35o	36o	41o	42o	43o	44o	45o	46o	51o
2	Label	UVA	UVA	UVA	UVA	UVA	UVA	UVA+CBD	UVA+CBD	UVA+CBD	UVA+CBD	UVA+CBD	UVA+CBD	UVB
3	PC(34:2)	33.14828	26.94982	34.39174	24.342	26.58079	30.59181	10.70796	9.132756	11.28927	8.705659	9.891374	13.41753	23.75
4	PC(38:4)	19.78598	16.08616	23.57916	27.57148	28.87821	19.22041	11.97407	9.849842	7.467128	9.735015	10.73764	13.69514	18.93
5	PC(36:4)	21.17842	17.21823	18.72432	15.5768	18.2788	11.87649	6.721466	6.705061	4.489629	5.464606	6.88567	8.209818	10.85
6	PC(36:2)	17.53081	14.25269	21.14495	11.94597	16.30759	22.58744	6.290938	4.17635	6.015601	5.114584	6.202147	6.205976	17.25
7	PC(36:3)	8.999805	7.316915	3.973059	6.289689	6.302422	6.89102	2.69604	1.707867	2.989224	2.191902	1.757356	1.990454	8.2
8	PC(34:1)	8.710135	7.081411	9.673774	5.94098	8.973093	5.70499	2.863173	3.053628	1.962558	2.327783	3.823368	2.719708	6.506
9	PCp(40:10	1.895047	1.540689	3.469478	0.960875	1.741747	2.122847	0.942874	0.756264	0.749503	0.766564	0.439946	0.618823	0.730
10	PC(38:6)	5.777246	4.696948	6.712903	3.718908	4.885186	6.536865	2.572692	2.252668	1.753437	2.091619	2.056087	2.504664	5.785
11	PC(40:6)	4.547674	3.697296	4.167215	3.256276	4.466495	4.47753	1.794969	1.810533	0.758692	1.459325	1.944066	1.629665	3.61
12	PC(38:5)	7.220989	5.870723	4.96746	3.96656	6.208818	4.398293	2.331693	1.704553	1.252162	1.895685	1.440191	2.217263	5.558
13	PC(38:3)	6.456182	5.248929	5.247377	4.713281	5.779728	5.804511	1.106925	1.281103	1.618487	0.899939	1.523886	1.977789	4.563
14	PCp(38:8)	0.458072	0.372416	3.321001	0.382332	1.576617	2.439797	0.889105	0.767987	0.07708	0.722849	0.625581	0.707743	0.084
15	PCp(46:11	2.001417	1.627169	1.46215	2.805471	2.644574	1.029084	0.944056	0.518654	0.957557	0.767525	0.531831	1.028246	2.151
16	PC(36:1)	2.487193	2.022108	2.647118	2.673555	2.4992	3.795296	0.92175	0.956716	0.725182	0.749391	0.698665	1.016985	2.659
17	PC(32:0)	0.853445	0.693858	1.983175	1.833962	1.950534	0.799554	0.504997	0.3721	0.269668	0.410567	0.511793	0.492068	1.338
18	PC(40:5)	1.658086	1.348037	2.203196	1.255584	1.341603	1.608074	0.784728	0.954855	0.257888	0.63799	0.739937	0.649707	1.423
19	PC(40:8)	1.196187	0.97251	1.105898	0.807384	0.769319	0.724417	0.408245	0.506945	0.258548	0.331907	0.319689	0.656387	0.721
20	PCp(40:9)	0.625875	0.508841	0.981443	0.041055	0.709243	1.11219	0.34927	0.250851	0.042363	0.28396	0.177259	0.184468	0.31
21	PC(40:7)	0.976195	0.793655	0.987512	0.403675	0.852949	0.514063	0.334917	0.250182	0.13351	0.27229	0.360807	0.255354	0.815
22	PCp(44:11	0.458761	0.372977	0.267901	0.425244	0.514786	0.345023	0.171912	0.174082	0.283493	0.139766	0.12482	0.145127	1.110
23	PCp(46:12	0.466495	0.379265	0.299791	0.487491	0.196159	0.192242	0.128268	0.124254	0.122989	0.104283	0.093287	0.096616	0.842
24	PC(34:0)	0.395871	0.321846	0.463346	0.872302	0.895371	0.594062	0.316818	0.407972	0.438596	0.257575	0.28748	0.387534	0.476
25	LPC(16:0)	56.41518	45.866	55.23348	72.01489	85.11445	82.41138	13.79757	13.0159	9.961463	11.21754	12.50427	18.67538	108.2
26	LPC(18:0)	66.0333	53.68561	35.12437	53.13466	78.10294	29.97122	8.223418	5.352836	7.080665	6.685705	6.958273	7.935559	32.03
27	LPC(18:2)	34.13928	27.75551	43.07199	46.95427	41.75653	58.41582	7.649823	7.730973	4.486279	6.219368	6.875186	4.497195	41.43
28	LPC(20:4)	27.63692	22.46904	18.66507	21.24294	33.12887	16.87548	1.554214	5.012596	4.860208	1.263588	5.330685	4.864097	18.71
29	LPC(18:1)	15.18421	12.34488	10.40648	11.63918	17.978	6.451567	2.516849	2.221299	1.973007	2.046219	1.897337	1.851026	8.681
30	LPC(22:6)	1.461675	1.188354	1.996312	0.960221	2.325631	1.88026	0.347838	0.401174	0.224535	0.282795	0.385683	0.359199	1.730
31	LPC(20:3)	2.386996	1.940647	1.863977	2.632198	2.415892	2.317427	0.335035	0.323333	0.409142	0.272386	0.340445	0.398172	2.946
32	LPC(16:1)	2.768395	2.250728	3.775068	2.314408	2.568495	1.591259	0.497615	0.335899	0.779089	0.404565	0.255808	0.382035	2.7
33	LPE(18:0)	2.076121	1.687904	2.611748	2.427076	2.636617	1.993515	0.849461	0.566246	0.893772	0.690619	0.950792	0.487645	9.343
34	LPE(16:0)	3.183569	2.588267	1.803002	1.615653	2.150394	1.202649	0.653083	0.941286	0.7592	0.530961	1.130581	1.656742	13.26
35	LPE(18:2)	2.002884	1.628361	1.575616	1.521757	2.771033	2.812783	0.869792	0.878183	1.069519	0.707148	1.144322	0.97244	8.774
36	LPE(20:4)	1.498818	1.218551	1.61606	0.893081	0.854318	0.630799	0.600054	0.720733	0.323347	0.487849	0.643216	0.555505	4.08
37	LPE(18:1)	1.241325	1.009208	1.067945	0.561731	1.558995	0.578202	0.535105	0.52379	0.425539	0.435045	0.638838	0.340417	2.424
38	LPE(22:6)	0.407647	0.33142	0.386184	0.34159	0.374717	0.35325	0.12137	0.122721	0.105533	0.098675	0.179772	0.098209	1.137
39	PE(38:4)	0.817262	0.664441	0.457032	0.220214	0.615747	1.074602	0.600298	0.771731	0.865628	0.488047	1.132112	0.800301	0.316
40	PE(36:2)	0.522432	0.424742	0.677658	0.290791	0.715858	0.622493	0.808511	0.805389	0.532004	0.657326	1.190014	0.595135	0.315
41	PE(36:4)	1.43392	1.165788	0.795147	0.237478	0.629695	0.481643	0.804612	0.533059	0.759722	0.654156	0.683425	1.071857	0.159

Table S3 Phospholipid species

Table S3. Ceramide molecular species (63) identified in the in the plasma of nude rats used in the present study (nonhydroxy fatty acid [N], dihydrosphingosine [DS], and sphingosine [S]).

CER Class	CER Specie		
	m/z	RT	Name
CER[NS]	580.5566	41.69	Cer(d18:1/19:0)
	552.5253	37.18	Cer(d18:1/17:0)
	648.6220	42.38	Cer(d18:1/24:1)
	636.6221	41.62	Cer(d18:1/23:0)
	622.6060	42.35	Cer(d18:1/22:0)
	634.6052	44.34	Cer(d18:2/23:0)
	650.6362	42.92	Cer(d18:1/24:0)
	594.5754	39.84	Cer(d18:1/20:0)
	594.5754	39.84	Cer(d16:1/22:0)
	592.5587	37.15	Cer(d18:2/20:0)
	620.5871	41.61	Cer(d18:2/22:0)
	578.5454	38.22	Cer(d15:2/22:0)
	566.5446	34.99	Cer(d18:1/18:0)
	664.6482	43.27	Cer(d18:1/25:0)
	524.4959	33.01	Cer(d16:1/17:0)
	608.5915	32.77	Cer(d16:1/23:0)
	590.5453	37.76	Cer(d18:2/20:1)
	676.6535	44.27	Cer(d18:1/26:1)
	606.5768	42.81	Cer(d18:2/21:0)

	564.5263	32.75	Cer(d18:1/18:1)
	538.5122	32.65	Cer(d18:1/16:0)
	536.4963	43.99	Cer(d18:2/16:0)
	678.6637	47.45	Cer(d18:1/26:0)
**CER[NDS]**	554.5405	37.44	Cer(d18:0/17:0)
	540.5259	33.56	Cer(d18:0/16:0)
	680.6856	30.41	Cer(d18:0/26:0)
	566.5446	34.99	Cer(d18:0/18:1)
	568.5581	38.40	Cer(d18:0/18:0)

Table S4. Peak area of each ceramide molecular species identified in the plasma of control rats (Ctr) and rats irradiated with UVA (increasing doses from 0.5 to 5 J/cm² for 4 weeks) or rats irradiated with UVB (increasing doses from 0.02 to 2 J/cm² for 4 weeks). These animals were not treated (Ctr, UVA, and UVB) or were treated topically with CBD (CBD, UVA+CBD, and UVB+CBD) (2.5 g CBD in 100 g petrolatum). Data obtained using MZmine software (XLSX).

	A	B	C	D	E	F	G	H	I	J	K	L	M
1	Name	1o	2o	3o	11o	12o	13o	21o	22o	23o	24o	25o	26o
2	Label	.Ctr	.Ctr	.Ctr	.Ctr	.Ctr	.Ctr	CBD	CBD	CBD	CBD	CBD	CBD
3	Cer(d18:1,	11.62426	8.135491	8.987558	9.734122	9.097209	15.4361	15.01989	14.63373	13.47437	13.77887	13.67499	14.48629
4	Cer(d18:1,	7.649239	6.019076	6.002317	7.078953	6.839227	13.32125	15.0881	13.69995	9.981318	11.58691	10.09603	10.5897
5	Cer(d18:1,	7.25452	10.64677	9.75454	9.262418	9.768737	14.2589	12.88261	12.13081	10.95769	11.56596	10.85961	10.39177
6	Cer(d18:1,	0.388286	0.807452	1.02637	0.872938	1.015711	1.265874	1.372659	3.076993	2.550706	1.617967	2.21546	1.82817
7	Cer(d18:1,	1.111631	1.035518	0.797489	0.825497	0.934621	0.805194	0.952223	2.334299	0.681841	1.024074	1.809735	0.763522
8	Cer(d18:2,	0.046175	0.093204	0.093844	0.071074	0.090439	0.235822	0.161495	0.802606	0.464565	0.575878	0.630529	0.59164
9	Cer(d18:1,	1.181001	1.799172	1.920438	1.251335	1.122367	1.108111	1.128936	1.1375	0.940059	0.997248	1.475255	0.882638
10	Cer(d18:1,	1.255198	0.767629	0.931328	0.604325	0.895632	0.707076	0.969971	0.825565	0.883453	0.534634	0.668771	0.44733
11	Cer(d16:1,	1.255198	0.767629	0.931328	0.604325	0.895632	0.707076	0.969971	0.825565	0.883453	0.534634	0.668771	0.44733
12	Cer(d18:2,	0.925617	0.833924	1.024789	1.088655	1.015376	0.472965	0.642569	0.64479	0.605558	0.46704	0.504686	0.550222
13	Cer(d18:2,	0.844869	1.027536	1.044441	1.113163	1.031892	0.602019	0.643037	0.766054	0.64595	0.601891	0.737789	0.532732
14	Cer(d15:2,	0.083642	0.043075	0.07653	0.061984	0.05483	0.029026	0.145212	0.120732	0.109767	0.161873	0.202718	0.146324
15	Cer(d18:1,	0.096527	0.165662	0.304809	0.207374	0.078942	0.130999	0.123134	0.203292	0.185235	0.266552	0.120722	0.156464
16	Cer(d18:1,	0.060493	0.048245	0.057495	0.032868	0.044582	0.050959	0.049324	0.092356	0.062966	0.071468	0.073865	0.081286
17	Cer(d16:1,	0.012897	0.010794	0.059464	0.011239	0.019863	0.053409	0.204807	0.323062	0.212093	0.244679	0.125795	0.18334
18	Cer(d16:1,	0.030839	0.012988	0.025376	0.032109	0.017035	0.018346	0.032647	0.067622	0.053476	0.057224	0.050122	0.060428
19	Cer(d18:2,	0.178403	0.071165	0.169815	0.066694	0.097142	0.219677	0.097406	0.095455	0.102628	0.148016	0.142543	0.160027
20	Cer(d18:1,	0.079141	0.147017	0.134034	0.148982	0.080204	0.054366	0.054801	0.056474	0.015368	0.253746	0.041201	0.16736
21	Cer(d18:2,	0.088056	0.056026	0.022077	0.041745	0.036674	0.121159	0.067808	0.073904	0.072879	0.091724	0.058746	0.061988
22	Cer(d18:1,	0.007566	0.014105	0.038701	0.015902	0.024511	0.015459	0.016341	0.063871	0.050404	0.066909	0.035512	0.0296
23	Cer(d18:1,	0.048837	0.014511	0.070551	0.045789	0.023755	0.028266	0.072348	0.072679	0.155169	0.037588	0.07399	0.034796
24	Cer(d18:2,	0.007876	0.002398	0.004023	0.001856	0.001736	0.006089	0.001806	0.001278	0.002677	0.001224	0.002168	0.00385
25	Cer(d18:1,	0.205937	0.260205	0.237428	0.383394	0.257679	0.31293	0.199809	0.256002	0.116401	0.079276	0.14306	0.127542
26	Cer(d18:0,	0.274117	0.157318	0.291927	0.308345	0.372376	0.417291	0.48793	0.474596	0.320878	0.383923	0.270977	0.398053
27	Cer(d18:0,	5.816059	4.39247	3.723813	5.600898	6.299752	3.50183	3.477915	4.471185	3.447409	3.488244	3.091348	2.062756
28	Cer(d18:0,	0.4153	0.187031	0.4378	0.242208	0.386725	0.436907	0.426909	0.300632	0.413192	0.354069	0.214041	0.287158
29	Cer(d18:0,	0.236046	0.178601	0.318956	0.224227	0.078942	0.130999	0.123134	0.203292	0.185235	0.266552	0.120722	0.156464
30	Cer(d18:0,	1.733122	0.905878	0.804107	1.230249	1.342864	2.394131	7.716897	6.039291	5.063963	4.314405	4.183016	3.608685

	A	N	O	P	Q	R	S	T	U	V	W	X	Y	Z
1	Name	31o	32o	33o	34o	35o	36o	41o	42o	43o	44o	45o	46o	51o
2	Label	UVA	UVA	UVA	UVA	UVA	UVA	UVA+CBD	UVA+CBD	UVA+CBD	UVA+CBD	UVA+CBD	UVA+CBD	UVB
3	Cer(d18:1,	58.85269	49.93842	48.30564	56.58019	32.82969	58.66906	51.13448	51.55836	55.72251	42.77842	52.45776	40.38687	82.09
4	Cer(d18:1,	43.40452	42.27	40.38274	46.5061	41.62539	53.47418	33.60893	38.98283	45.16028	31.16958	44.58636	22.54558	57.7
5	Cer(d18:1,	46.44171	45.58614	41.49969	42.343	47.5092	50.80005	43.57766	42.69519	48.46105	35.20489	48.81669	39.4101	66.58
6	Cer(d18:1,	4.945038	5.312668	6.667637	11.92274	9.983164	7.385063	8.293699	6.112448	9.390202	6.537943	8.11347	11.18589	7.657
7	Cer(d18:1,	3.54225	1.902048	4.011505	5.292147	2.752076	3.409727	3.347816	3.258527	3.217286	2.589437	3.162535	5.05626	3.343
8	Cer(d18:2,	1.037223	1.46342	2.100344	2.149992	1.929466	1.133419	1.179221	2.596985	0.808809	0.91118	2.682817	2.247537	3.30
9	Cer(d18:1,	3.859802	3.657642	3.352913	3.731923	3.861274	4.536818	3.535554	3.358673	4.043453	2.784467	4.107981	3.164308	5.275
10	Cer(d18:1,	2.914317	2.196181	2.279882	3.075599	3.252096	3.775086	2.94333	2.052017	2.015204	1.994718	2.672362	2.089311	3.267
11	Cer(d16:1,	2.914317	2.196181	2.279882	3.075599	3.252096	3.775086	2.94333	2.052017	2.015204	1.994718	2.672362	2.089311	3.267
12	Cer(d18:2,	1.939247	1.129031	1.92366	1.959216	1.888306	2.56881	1.815982	2.207036	1.403373	1.880736	2.205247	1.722829	2.050
13	Cer(d18:2,	1.251085	2.108829	1.929832	1.68479	2.404248	1.638603	1.265518	1.52798	2.355086	1.099261	2.486304	1.411292	3.056
14	Cer(d15:2,	1.186147	0.183648	0.484681	0.804425	0.518314	0.719274	0.354332	0.609353	0.392085	0.188819	0.331734	0.611617	0.83
15	Cer(d18:1,	1.171448	1.282656	1.176023	1.482169	1.086405	1.615941	1.268371	1.198753	1.065941	1.371777	1.518036	1.289972	1.318
16	Cer(d18:1,	4.277184	6.611645	6.151194	12.93285	12.09134	5.425137	6.396565	4.591862	9.559628	4.062732	0.285715	0.174575	13.74
17	Cer(d16:1,	1.345407	1.283905	0.657517	1.320049	0.762308	1.051216	0.658796	0.873893	0.995713	0.726658	1.056667	0.408297	1.466
18	Cer(d16:1,	0.195442	0.058606	0.14664	0.063655	0.083928	0.164053	0.081046	0.209741	0.231778	0.074103	0.08146	0.233	0.205
19	Cer(d18:2,	0.25428	0.224579	0.481043	0.234168	0.297869	0.213642	0.761081	0.581995	0.62087	0.512632	0.376224	0.60115	0.484
20	Cer(d18:1,	0.139975	0.157583	0.18803	1.528353	0.306188	0.263358	0.507784	0.675477	0.180978	0.639374	0.164416	0.184533	0.507
21	Cer(d18:2,	0.388998	0.281331	0.292003	0.807293	0.269732	0.452427	0.258353	0.218399	0.429738	0.22761	0.333492	0.275993	0.472
22	Cer(d18:1,	0.32528	0.298894	0.19509	0.315926	0.201989	0.22777	0.171361	0.214601	0.228041	0.182093	0.229294	0.103452	0.308
23	Cer(d18:1,	0.09476	0.156832	0.457984	0.476517	0.22263	0.188301	0.653344	0.525033	0.191627	0.140638	0.143019	0.224695	0.839
24	Cer(d18:2,	0.014151	0.007383	0.028038	0.008123	0.009857	0.009991	0.003095	0.009092	0.010013	0.003095	0.00474	0.009148	0.028
25	Cer(d18:1,	1.109146	1.166699	1.245286	1.142001	1.135077	1.2295	1.448888	1.659591	0.722376	1.418106	1.07959	1.979653	1.886
26	Cer(d18:0,	2.387921	2.31272	2.331158	1.38347	1.328313	1.463094	1.225003	1.226161	1.392787	1.236607	1.300901	1.193097	2.395
27	Cer(d18:0,	35.37427	23.83185	31.75234	35.88916	33.87854	32.88428	35.11092	17.79344	25.32385	17.03022	19.47883	21.20143	35.44
28	Cer(d18:0,	1.639221	1.392038	1.559152	1.199281	1.489182	2.049943	1.488215	1.7839	1.411959	1.051249	1.160976	1.811442	1.681
29	Cer(d18:0,	1.607582	2.885977	1.71051	7.83488	1.944121	1.385867	0.951278	2.221933	2.398367	0.836498	1.165582	2.024363	1.318
30	Cer(d18:0,	21.96445	8.30368	14.31165	9.084843	17.22033	21.05385	23.10031	22.17474	23.02981	22.40714	21.93936	22.17691	20.65

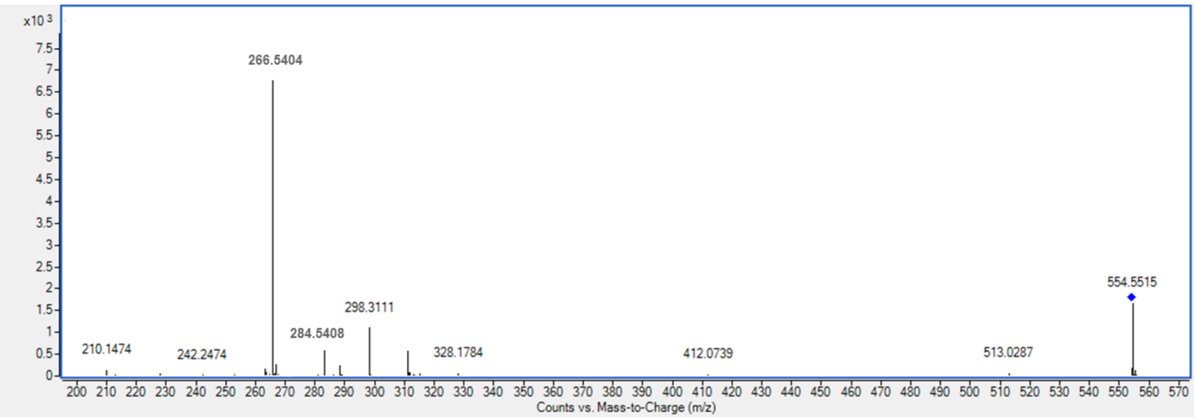

Figure S1. ESI-MS/MS spectrum of the $[M+H]^+$ ion of Cer(d18:0/17:0) [m/z 554.5515; RT 37.44 min]. Characteristic fragment ions are: m/z 266.5404 corresponding to $[M+H]^+$-FA-2H_2O and m/z 284.5408 corresponding to $[M+H]^+$-FA-H_2O.

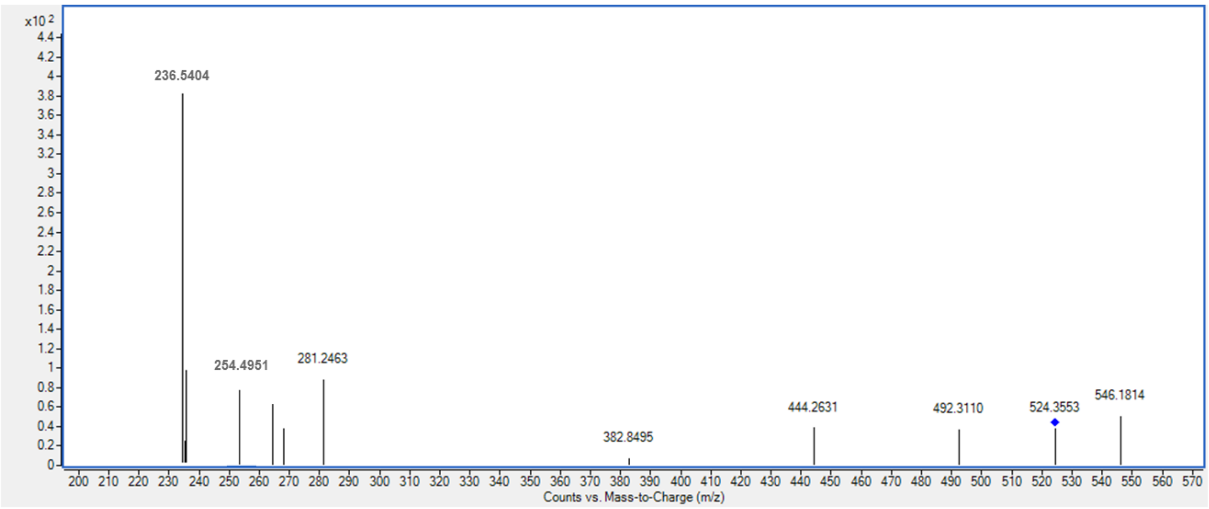

Figure S2. ESI-MS/MS spectrum of the [M+H]$^+$ ion of Cer(d16:1/17:0) [m/z 524.3553; RT 33.02 min]. Characteristic fragment ions are: m/z 236.5404 corresponding to [M+H]$^+$-FA-2H_2O and m/z 254.4951 corresponding to [M+H]$^+$-FA-H_2O.

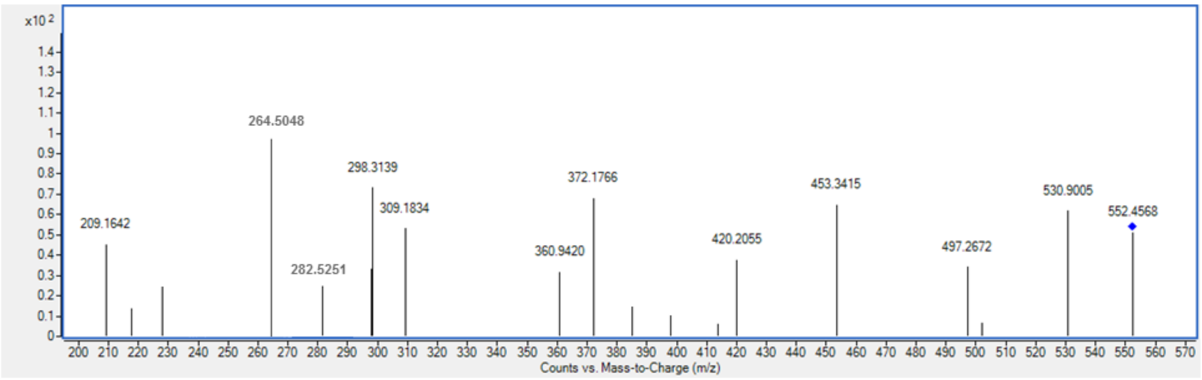

Figure S3. ESI-MS/MS spectrum of the [M+H]$^+$ ion of Cer(d18:1/17:0) [m/z 552.4568; RT 37.18 min]. Characteristic fragment ions are: m/z 264.5048 corresponding to [M+H]$^+$-FA-2H_2O and m/z 282.5251 corresponding to [M+H]$^+$-FA-H_2O.

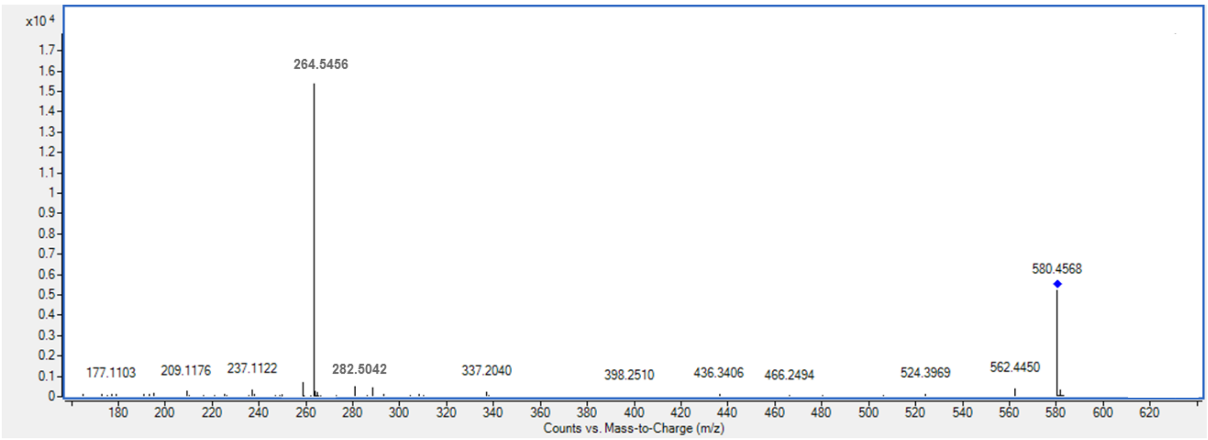

Figure S4. ESI-MS/MS spectrum of the [M+H]$^+$ ion of Cer(d18:1/19:0) [m/z 580.4568; RT 41.68 min]. Characteristic fragment ions are: m/z 264.5456 corresponding to [M+H]$^+$-FA-2H_2O and m/z 282.5042 corresponding to [M+H]$^+$-FA-H_2O.
